# 

**Published:** 1895-03

**Authors:** 


### CONTENTS—MARCH, 1895.—VOL. X., NO. 9.

ORIGINAL COMMUNICATIONS—

Texas Asylums in Politics. James Orr, M. D. . . . . . . . . . . . 445
Medical Fads and Faddists. LeRoy Dibble, M. D., Kansas City, Mo. 449
Treatment of Traumatic Cataract. James Moores Ball, M. D.. St. Louis, Mo . . . . . . . . . . . . . . . . . . . . . . . . . . . . . 454
The Management of Chronic Invalids. G. W. Christian, M. D., Houston, Texas. . . . . . . . . . . . . . . . . . . . . . . . . . . 456

SOCIETY NOTES—

Proceedings of Terrell Medical Society . . . . . . . . . . . . . . 460
Houston District Medical Society . . . . . . . . . . . . . . . . . . 461
The American Sanitary Association . . . . . . . . . . . . . . 462
American Medical Publishers' Association . . . . . . . . . . . . . . 462

ABSTRACTS AND SELECTIONS—

A New Operation for the Radical Cure of Inguinal and Femoral Hernia . . . . . . . . . . . . . . . . . . . . . . . . . . 463
The Diphtheria Cure. . . . . . . . . . . . . . . . . . . . . . . . . 467

EDITORIAL—

The Antitoxine Treatment of Diphtheria . . . . . . . . . . . . . . 469
The Small-Pox Outbreak . . . . . . . . . . . . . . . . . . . . 474
Asylums in Politics . . . . . . . . . . . . . . . . . . . . . . . . . 476
Rape in Texas—Castration the Only Remedy. . . . . . . . . . . 477
The Milk in the Cocoanut.. . . . . . . . . . . . . . . . . . . . . 479
To "Regulate the Practice" . . . . . . . . . . . . . . . . . . . . . 480
Pollution of Streams . . . . . . . . . . . . . . . . . . . . . . . . 481

PROGRESS IN MEDICINE .

Abortion of Small-Pox.. . . . . . . . . . . . . . . . . . . . . . . 482
The Use of Horse Hair in Surgery.. . . . . . . . . . . . . . . . . . 486

MEDICAL NEWS AND MISCELLANY—

Items of General Information. . . . . . . . . . . . . . . . . . . . . 488

BOOK NOTICES— . . . . . . . . . . . . . . . . . . . . . . . . . . 493
PUBLISHERS' NOTES . . . . . . . . . . . . . . . . . . . . . . . . 500

